# Drought and shade deplete nonstructural carbohydrate reserves in seedlings of five temperate tree species

**DOI:** 10.1002/ece3.1819

**Published:** 2015-11-19

**Authors:** Andrea J. Maguire, Richard K. Kobe

**Affiliations:** ^1^ Department of Plant BiologyMichigan State UniversityEast LansingMichigan; ^2^ Graduate Program in Ecology, Evolutionary Biology and BehaviorMichigan State UniversityEast LansingMichigan; ^3^ Department ForestryMichigan State UniversityEast LansingMichigan

**Keywords:** Carbon reserves, carbon starvation, nonstructural carbohydrates, soluble sugars, starch, stress

## Abstract

Plants that store nonstructural carbohydrates (NSC) may rely on carbon reserves to survive carbon‐limiting stress, assuming that reserves can be mobilized. We asked whether carbon reserves decrease in resource stressed seedlings, and if NSC allocation is related to species' relative stress tolerances. We tested the effects of stress (shade, drought, and defoliation) on NSC in seedlings of five temperate tree species (*Acer rubrum* Marsh., *Betula papyrifera* Marsh*., Fraxinus americana* L *., Quercus rubra* L., and *Quercus velutina* Lam.). In a greenhouse experiment, seedlings were subjected to combinations of shade, drought, and defoliation. We harvested seedlings over 32–97 days and measured biomass and NSC concentrations in stems and roots to estimate depletion rates. For all species and treatments, except for defoliation, seedling growth and NSC accumulation ceased. Shade and drought combined caused total NSC decreases in all species. For shade or drought alone, only some species experienced decreases. Starch followed similar patterns as total NSC, but soluble sugars increased under drought for drought‐tolerant species. These results provide evidence that species deplete stored carbon in response to carbon limiting stress and that species differences in NSC response may be important for understanding carbon depletion as a buffer against shade‐ and drought‐induced mortality.

## Introduction

Tree mortality has been increasing worldwide due to environmental stressors including drought, pest‐outbreaks, and increases in temperature (Allen et al. 2010; Williams et al. 2010; Carnicer et al. 2011; Choat et al. 2012). These trends motivate a strong interest in understanding the mechanisms behind tree mortality and the interactions among different types of stress that could inform predictions of tree responses to global change in the future (Fisher et al. 2010). Carbon reserves are hypothesized to play a role in the tolerance of environmental stress, especially when carbon gain is limited (McDowell et al. 2008; Anderegg et al. 2012; O'Brien et al. 2014). Carbon gained through photosynthesis can be allocated to growth, reproduction, defense, and maintenance of metabolic functions. Assimilated carbon also can be stored for later use as nonstructural carbohydrates (NSC), mainly consisting of starch, sucrose, glucose, and fructose (Chapin et al. 1990; Kozlowski 1992). Storage could have several adaptive advantages as NSC may be mobilized for future growth, recovery of lost tissue, and fueling respiratory needs (Chapin et al. 1990; Kozlowski 1992; Hoch et al. 2003). Carbon balance is vital to plant performance, and stress‐induced NSC depletion could be a mechanism underlying tree mortality (McDowell et al. 2008; Breshears et al. 2009; Sala et al. 2012; Dietze et al. 2014).

The role of NSC reserve depletion as a mechanism underlying tree mortality has been debated, especially for cases of drought stress (Adams et al. 2009; McDowell 2011; Sala et al. 2012). A synthesis of mortality mechanisms suggests two related hypotheses of drought‐induced mortality: carbon starvation and hydraulic failure (McDowell et al. 2008). Drought can have direct consequences such as embolism, hydraulic failure, and cell failure (Bréda et al. 2006), but it can also affect a tree's carbon balance. Drought also can impede photosynthesis through leaf loss and stomatal closure, causing the tree to increase reliance on stored NSC to meet its metabolic demand. When carbon is no longer available to sustain basic functions, a tree may die of carbon starvation (McDowell et al. 2008; Breshears et al. 2009; Adams et al. 2013). On the other hand, drought could decrease a tree's carbon demand by downregulating growth, leading to a sink limitation, and in some cases, increased allocation to storage (Sala et al. 2012). When NSC has been measured directly, results have provided mixed support for the role of NSC reserve mobilization, including examples of NSC depletion (Galiano et al. 2011; Adams et al. 2013; Mitchell et al. 2013; increased NSC storage (Galvez et al. 2011; Muller et al. 2011; Anderegg et al. 2012), and no NSC response (Gruber et al. 2011). Starch and other NSC forms also could also be converted to simple sugars for other purposes such as to maintain osmotic potential, without a depletion of total NSC (O'Brien et al. 2014). Carbon starvation due to defoliation has similarly conflicting results. After defoliation, NSC has been shown to both decrease and increase and can depend on refoliation (Eyles et al. 2009; Piper et al. 2009; Landhäusser and Lieffers 2011; Machado et al. 2013). Different types of stress can have complex interactions with carbon balance and the co‐occurrence of different types of stress can increase the complexity of the NSC response. For example, shade or defoliation could lessen the impact of drought by decreasing the loss of water through stomata (Sack and Grubb 2002).

Species differences in responding to environmental stresses may lead to contradictory results for the role of NSC in stress tolerance. For drought tolerance, species differences in the regulation of water loss could result in differences in the risk of carbon starvation versus hydraulic failure (McDowell et al. 2008), and the two mechanisms likely interact (McDowell 2011; Sevanto et al. 2014). Species differences in adaptation to shade also may affect the role of NSC in shade tolerance. Trees are known to exhibit a growth‐survival trade‐off where species that tolerate shade tend to grow slowly and allocate more photosynthate to NSC storage versus those that are shade intolerant favor rapid growth and allocate more to structural growth (Kobe 1997; Myers and Kitajima 2007; Poorter and Kitajima 2007). Similarly, species differences in NSC storage are related to differences in survivorship in environments with low water availability (Meier and Leuschner 2008) and after leaf tissue loss (Canham et al. 1999; Poorter and Kitajima 2007). Correlations between species survival and NSC storage suggest that NSC can act as a buffer that protects against multiple low carbon‐limiting stressors (Chapin et al. 1990; Kitajima 1994; Kobe 1997; Canham et al. 1999). If a species' NSC storage enhances survival during times of stress, then increased NSC storage could either buffer against multiple stresses or for different stresses depending on the species' traits (Chapin et al. 1993). To obtain a fuller picture of how and if stored carbon is depleted under carbon limitation, we explore the interactions of multiple stressors and how species with varying tolerance may respond differentially.

We examined the NSC response to stress in seedlings of five temperate tree species from a range of shade and drought tolerances. Different combinations of defoliation, deep shade, and severe drought stress were imposed to induce varying degrees of carbon source and sink limitation. NSC response was measured over time in seedlings under each treatment. If stored NSC is driving survivorship differences among and within species under different environmental stresses, then NSC should be mobilized under stress. We hypothesized that shade, drought, and defoliation are carbon limiting and negatively impact plant carbon balance (Hypothesis 1) and that seedlings will rely on stored carbon to survive carbon limitation and therefore deplete their stored carbon reserves over time (Hypothesis 2). We further hypothesize that seedling NSC response to shade and drought will differ depending on the species stress tolerance rankings such that the more stress tolerant species will maintain their carbon reserves due to higher initial storage or a smaller depletion rate (Hypothesis 3).

## Materials and Methods

### Study species

**Figure 1:**
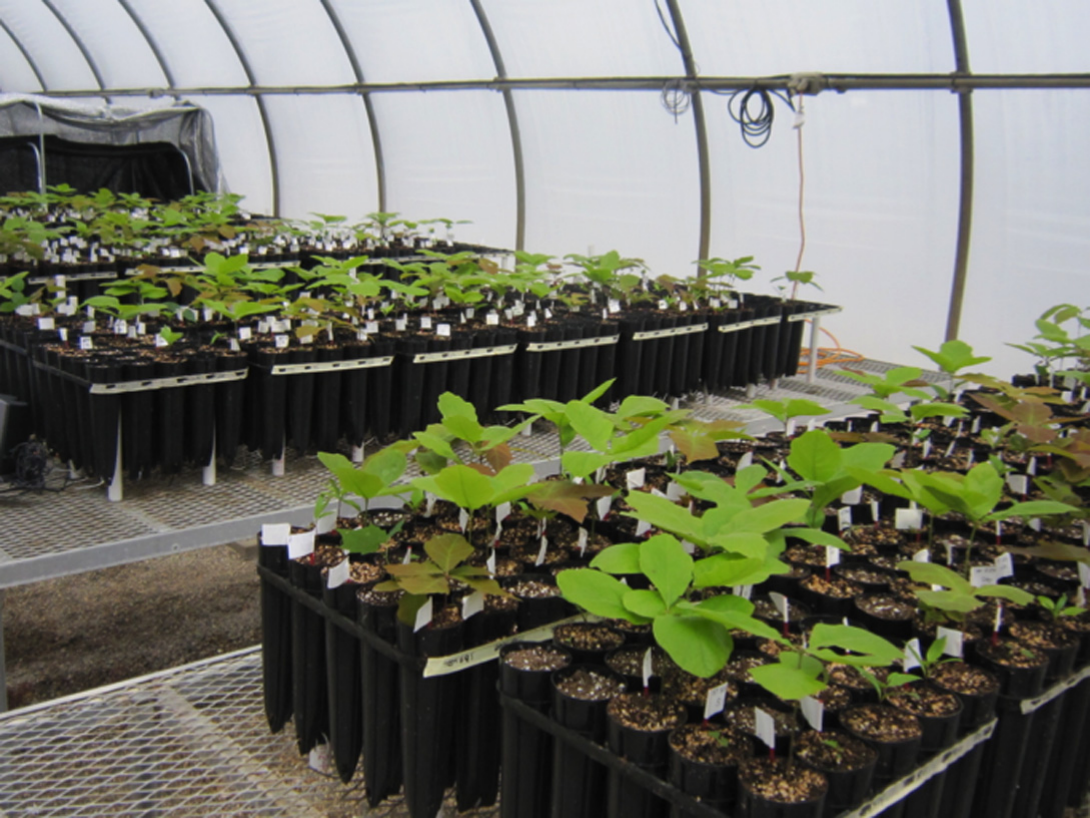
Seedlings growing in the greenhouse.

This study included five northern hardwood tree species with a range of shade and drought tolerances: *Acer rubrum* Marsh. (AR), *Betula papyrifera* Marsh. (BP), *Fraxinus americana* L. (FA), *Quercus rubra* L. (QR), and *Quercus velutina* Lam. (QV) (Table 1). These species are common in deciduous forests throughout eastern North America. The experiment was conducted in a greenhouse at Michigan State University's Tree Research Center in East Lansing, MI from April to November 2010 (Fig. 1). Seed was obtained from a commercial seed source (Sheffield Seed Co., Locke, NY) for *A. rubrum*,* B. papyrifera*,* Q. rubra*, and *Q. velutina*. Seedlings for *F. americana* were collected from the field as new germinants and transplanted into pots at the greenhouse because the length of the seed stratification period was prohibitive. The mass of each seed was measured after removal of accessory structures (e.g., wings and acorn caps) for *A. rubrum*,* Q. rubra*, and *Q. velutina*.

**Table 1:** Study species and their relative shade and drought tolerance based on Burns and Honkala (1990)

Species	Common name	Shade tolerance	Drought tolerance
*Acer rubrum* (AR)	Red maple	Tolerant	Intermediate
*Betula papyrifera* (BP)	Paper birch	Very intolerant	Very intolerant
*Fraxinus americana* (FA)	White ash	Intermediate	Intermediate
*Quercus rubra* (QR)	Northern red oak	Intermediate	Tolerant
*Quercus velutina* (QV)	Black oak	Intolerant	Very tolerant

### Experimental design

All seeds were planted in individual 660 mL pots under moderately high light levels (about 50% full sun) and watered as needed to maintain soil moisture during the establishment phase before treatments were applied. Seeds were planted in a commercial mixture (Fafard #2; BFG Supply Co., Kalamazoo, MI) that included basic nutrients, plus field soil with a volume ratio of 10:1 commercial mixture: field soil (Kobe et al. 2010). This combination allowed for ease of harvest while still including natural soil microbes. Field soil was obtained from the Manistee National Forest in northern lower Michigan and at the Tree Research Center's Sandhill Research Forest in East Lansing, MI. Seedlings were occasionally sprayed (Avid 0.15 EC Syngenta Crop Protection, Inc. Greensboro, NC; Tame 2.4 EC, Valent U.S.A, Co., Walnut Creek, CA; Terraguard, OHP, Inc., Mainland, PA) for common greenhouse pests. After an initial growth period to allow a sufficient number of seedlings to establish (12−22 weeks), seedlings were submitted to a 2 × 2 × 2 factorial experiment: (<3% vs. 50% full sun) × (drought vs. watering as needed) × (50% defoliation vs. no defoliation) for a total of eight treatment combinations. *Acer rubrum* seedlings had a longer establishment period than the other species due to low numbers of successful germinants and re‐planting. Our control treatment was the same as prestress conditions (50% sun, watering as needed, and no defoliation). For the shade treatment, benches were covered with two layers of shade cloth each (80% and 70% light reduction), reducing light to <3% full sun; light levels were verified with a quantum sensor. The drought treatment consisted of no watering. In the defoliation treatment, we simulated herbivory by a one‐time removal of whole leaves at the base of the petiole, alternating sides when possible, until approximately 50% of the total leaf area was removed. Seedlings of each species were randomly assigned to each treatment.

### Harvests

We began surveying seedlings for survivorship once treatments started; however, there was no observed mortality based on visual assessment of presence green of tissue. Before treatments were imposed, 24 seedlings per species were harvested to establish pretreatment biomass and NSC levels. Then, three seedlings per species in each treatment were harvested at several time points over a period of 32–97 days resulting in about six harvest per species for a total of 885 seedlings. The harvest schedule was based on expectations of survival times for the different species – treatment combinations, as well as logistics of harvesting with the aim of getting six time points throughout the harvest period. We achieved at least six harvests for all species except *A. rubrum*, which were harvested over the shortest time frame and only had five harvests due to lower seedling numbers from the start. After harvesting, root systems were carefully hand washed with water and a sieve, and tissue was separated into stems, leaves, and roots. Stem and root samples were microwaved for 60 sec at 600 W to denature enzymes that could degrade NSC molecules. Tissues were put in drying ovens overnight at 65°C, and dry mass was measured for each component.

### NSC analysis

Nonstructural carbohydrate concentrations were determined from stem and root samples for each seedling harvested. Dried samples were homogenized and pulverized to a fine powder using a ball mill (Kinetic Laboratory Equipment, Visalia, CA) and stored at 4°C until analysis. Small samples were ground by hand using a mortar and pestle. NSC was measured in two steps modified from Kobe et al. (2010). First, we extracted soluble sugars from 12 to 14 mg samples three times in 80% ethanol by heating and then centrifuging for 5 min at 1900 *g*. Concentrations in the supernatant were measured using a phenol‐sulfuric acid colorimetric assay (DuBois et al. 1956). The remaining pellet was put in a steam bath for 1 h to gelatinize the starch and then incubated with amyloglucosidase (Sigma‐Aldrich Corp., St. Louis, MO) at 55°C for 16 h to digest the starch. The digested sample was analyzed colorimetrically using a glucose hexokinase assay reagent (G3293 Sigma‐Aldrich Corp.) and read on an absorbance microplate reader (ELx808 Absorbance Microplate Reader; BioTek Instruments, Inc., Winooski, VT). Total NSC concentrations were calculated as the sum of soluble sugar and starch concentrations derived from the assays. Total NSC pools were calculated as the product of sample NSC concentration and total dry mass of the organ.

**Figure 2:**
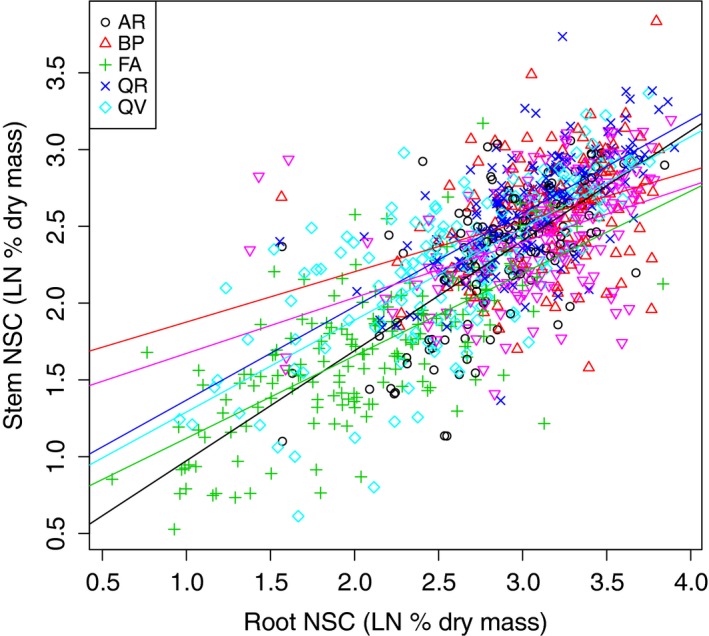
Relationship between the ln of stem and root nonstructural carbohydrate (NSC) concentrations for five temperate tree species.

### Data analysis

The change in root and stem NSC over time was analyzed by fitting linear models of NSC concentration as a function of time for each species‐treatment combination. We used a maximum‐likelihood approach to estimate the starting point and rate of change of NSC levels. Data were transformed (ln (NSC + 1)) prior to analysis in order to normalize the data with positive numbers. For each species, we tested models for all NSC components: a null model, a model with only time as a covariate, and ten models with time and different treatment combinations. To test for the effects of each treatment, we compared models with all eight treatments (control [C], drought [D], defoliation [H], defoliation + drought [HD], shade [S], shade + defoliation [SH], shade + drought [SD], shade + defoliation + drought [SHD]) to models that collapsed the focal treatment into the others. For example, pooling defoliation into the other treatments results in four possibilities (C, D, S, and SD). In all models that included treatments, we estimated a common intercept that represents the NSC starting point at the first harvest before treatments were applied. To compare whether treatments had an effect, we used AICc to choose the best supported model. The best model was considered to be the simplest model within two units of the minimum AICc (Burnham and Anderson 2002). For all species and most NSC components (39 of 45), the best common model pooled defoliation with other treatments. To facilitate comparisons among species, we used this model in all cases.

Slope estimates represent the rate at which seedlings deplete NSC reserves. Estimates were considered significant when 95% support intervals did not encompass zero. We also examined correlations between soluble and starch NSC concentrations in both the stem and root components to understand stress‐induced allocation responses. Growth was analyzed by fitting linear models of biomass change over time. We also compared pretreatment species means for NSC and biomass components using ANOVA. For *F. americana*, mass for the first harvest was mistakenly not recorded before seedlings were ground, so it was estimated from the *y*‐intercept (i.e., zero time). We estimated uncertainty by constructing confidence intervals using the support intervals from the slope estimates. These patterns were related back to expected growth and survival from the literature and the different life‐history strategies for each species (Table 1). All analyses were completed in R version 2.12.1 (R Development Core Team 2010), using the package bbmle (Bolker 2012).

## Results

### Pretreatment biomass and NSC concentrations

Initial mean biomass differed among species (QV^a^ > QR^b^ > BP^b^ > FA^b^ > AR^c^), as did root mass fraction (QV^a^ > QR^b^ > FA^c^ > AR^d^ > BP^d^). Pretreatment levels of NSC varied among species with mean concentrations ranging from 7.12 to 22.97% dry mass. Mean concentrations of NSC were highest in the species with the highest root mass fraction and had similar relative rankings (QV^a^ > QR^b^ > AR^b^ > FA^b^ > BP^c^) (Table 2). When looking at the separate tissues, the rankings differed slightly, but all species had higher concentrations in the root than in the stem (Table 2). Root NSC concentrations were an average of 1.35–2.9 times higher than stem NSC (Table 2 Fig. 2).

**Table 2:** Means and standard deviations for mass and nonstructural carbohydrate (NSC) components of seedlings sampled before treatment. a) NSC concentration, b) Starch concentration, c) Soluble sugar concentration d) Plant mass and age. Species abbreviations in Table 1. * Values in gray for FA were estimated from linear models because total plant mass was not measured for the first harvest and we could not calculate them directly

	Total		Stem		Root	
	Mean	SD	Mean	SD	Mean	SD
(a) NSC (% dry mass)						
AR	15.10	6.14	10.61	4.79	18.40	7.42
BP	7.12	1.66	5.93	2.02	9.16	2.25
FA	17.93	4.15	14.31	4.39	19.29	5.02
QR	14.94	4.21	8.62	2.11	16.82	5.03
QV	22.97	3.49	9.07	1.66	26.23	3.86
(b) Starch (% dry mass)						
AR	10.68	5.46	6.74	4.23	13.43	6.72
BP	1.58	0.71	0.52	0.65	3.45	1.73
FA	10.14	3.45	8.54	3.87	11.38	3.81
QR	11.05	3.81	4.45	2.27	13.06	4.58
QV	20.10	4.00	4.89	1.89	23.68	4.45
(c) Soluble Sugar (% dry mass)						
AR	4.42	1.01	3.86	1.40	4.98	1.27
BP	5.54	1.35	5.40	1.80	5.72	1.21
FA	7.80	1.49	5.98	1.47	8.68	1.72
QR	3.89	0.87	4.17	1.01	2.55	1.05
QV	2.87	0.86	4.18	1.06	2.55	0.98

	Mass (mg)		Root mass Fraction		Age (days)	
	Mean	SD	Mean	SD	Mean	SD
(d) Mass and Age						
AR	1989	1377	0.31	0.11	131	15
BP	3530	832	0.20	0.03	101	0
FA	3370	**–**	0.50	**–**	87	1
QR	3802	1772	0.56	0.11	87	3
QV	5026	1131	0.61	0.08	98	1

AR, *Acer rubrum* Marsh; BP, *Betula papyrifera* Marsh.; FA, *Fraxinus americana* L.; QR, *Quercus rubra* L.; QV, *Quercus velutina* Lam)

Starch made up most of the NSC, except in *B. papyrifera,* which had higher concentrations of soluble sugars. Mean starch across species ranged from 22.2 to 87.5% of the total NSC, with average concentrations from 1.58 to 20.1% dry mass. Starch tended to be located mostly in the root, with an average of 1.33–4.8 times more starch in roots than stems across species (Table 2). Concentrations of soluble sugars ranged from 2.87 to 7.8% dry mass. Soluble sugars were highest in *F. americana* and *B. papyrifera*, the species that had the lowest starch, and lowest in *Q. velutina*, which had the highest starch. *A. rubrum*,* B. papyrifera,* and *F. americana* had higher soluble sugar concentrations in the root and *Q. velutina* and *Q. rubra* in the stem, but the differences were not significant. Soluble sugars tended to be more evenly distributed among stems and roots than starch (Table 2).

The initial NSC concentration was correlated with seed size within species for *Q. velutina* (*r *=* *0.79, *P *<* *0.001), but not *Q. rubra* (*r *=* *0.26, *P *=* *0.274) or *A. rubrum* (*r *=* *0.26, *P *=* *0.239). Among species, the largest seeded species (*Q. velutina*) had the highest pretreatment mass and NSC concentration, and the smallest seeded species (*B. papyrifera*) had the lowest starting point of NSC, although not the lowest mass (Table 1). Initial NSC concentrations were also related to shade and drought tolerance levels of the species, although did not directly follow rankings. The least shade tolerant *B. papyrifera* had the lowest NSC, the most shade tolerant *A. rubrum* had intermediate NSC. NSC was greater for the drought‐tolerant oaks, and lower for drought intolerant *B. papyrifera* (Tables 1 and 2).

### Changes in the control treatment over time

**Figure 3:**
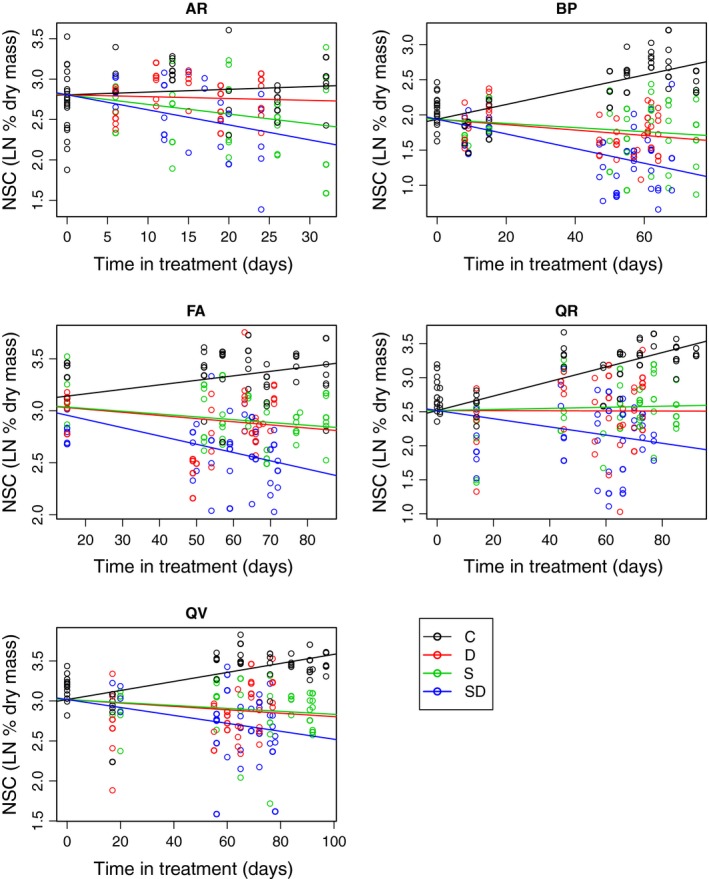
Total nonstructural carbohydrate (NSC) concentrations in seedlings over time under five treatments: C = control (50% light, well‐watered), D = drought (50% light and no water), S = shade (<3% light, well‐watered), SD = shade + drought (<3% light, no water). Each point represents one seedling. Lines are best‐fit linear models for each treatment with a common intercept, and NSC concentrations are natural log transformed. Models include seedlings from a nonsignificant defoliation treatment that is pooled with other treatments.

The seedlings in the control treatments increased in biomass over time for all species (Fig. S2), with root mass fraction also increasing in all species. As biomass increased, controls accumulated NSC (total pool size) in all species. NSC concentrations based on plant mass (% dry mass) also increased in all species (Fig. 3), but was not significant for *A. rubrum*. NSC pools were strongly positively correlated with total mass (Fig. S1). The rate of increase for NSC concentration was higher in *Q. rubra* and *B. papyrifera* than the other species (QR^a^ > BP^a^ > FA^b^ > QV^b^). The NSC increase was in both the stem and the root for all species except *A. rubrum* (Figs. 2 and S3).

### Effects of stress treatment on biomass over time

Seedlings ceased growth in every stress treatment, and lost biomass for all treatments in *B. papyrifera* and *Q. velutina*. Root mass fraction either increased or stayed the same in all the stress treatments except for a decrease in the shade + drought treatment for *Q. rubra*. The drought treatment had increased root mass fraction in all species except for *A. rubrum*, but the shade + drought treatment only had increases for *A. rubrum*. The shade treatment had increases in root mass fraction for *A. rubrum*,* F. americana,* and *Q. velutina*. The increased root mass fraction was due more to leaf loss than increases in root biomass; there were no significant increases in root biomass in any of the stress treatments, and only one case of decreased stem mass (Fig. S2).

### Effects of stress treatment on NSC concentrations over time

The defoliation treatment had little effect on NSC concentrations, with results similar to the control; thus, all treatments that included defoliation were grouped with other treatments. Collapsing the defoliation treatment was supported by model comparisons; in a majority of cases, the best model by AICc included three treatments (drought, shade, shade + drought) and the control. All results are reported for these groupings. As predicted, NSC concentrations in the stress treatments either stopped increasing or decreased over time (Figs. 3 and S3).

Nonstructural carbohydrates decreased most sharply with multiple stresses; the shade + drought treatment decreased NSC in all species. In shade, NSC decreased for *A. rubrum*,* F. americana,* and *B. papyrifera*. Under drought, total NSC decreased for *B. papyrifera*, and *F. americana*, while starch decreased in all species. Otherwise, starch patterns were similar to total NSC (Fig. S5). Soluble sugars did not follow the same trends as total NSC. There were increases in soluble sugars in the drought treatment for the drought‐tolerant species *A. rubrum*,* Q. rubra,* and *Q. velutina* (Fig. 3), which explains lack of decrease of total NSC in these species despite decreases in starch. Soluble sugars decreased in the shade treatment for *B. papyrifera* but not for other species. The shade + drought treatment had both increases (*Q. rubra* and *Q. velutina*) and decreases (*B. papyrifera*, and *F. americana*). Whenever soluble sugars changed, the change was in both stem and root tissue (Fig. S5).

**Figure 4:**
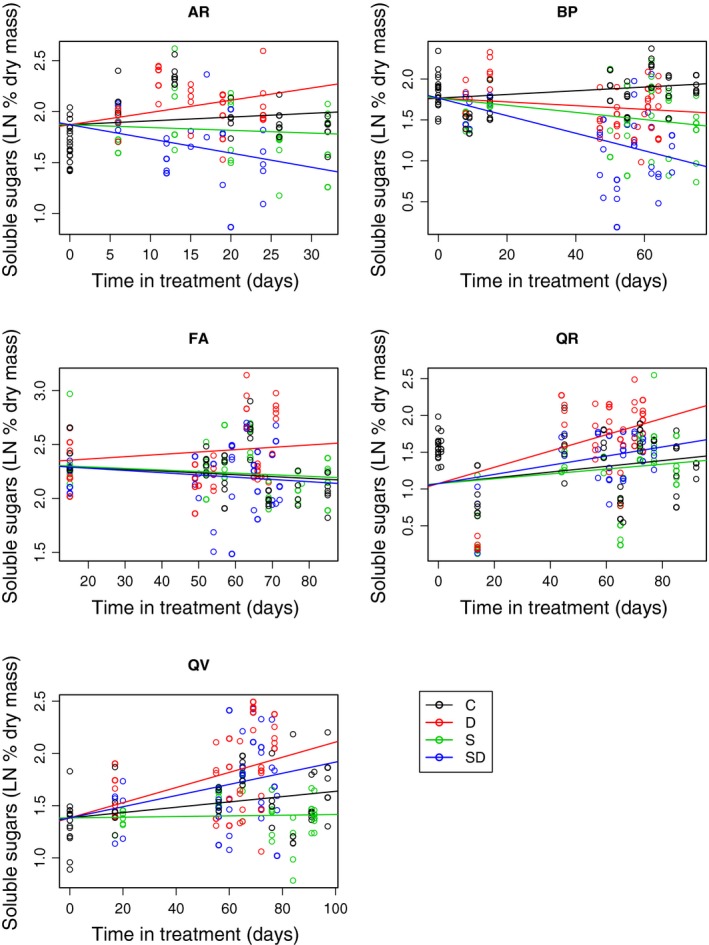
Soluble sugar concentrations in seedlings over time under five treatments: C = control (50% light, well‐watered), D = drought (50% light and no water), S = shade (<3% light, well‐watered), SD = shade + drought (<3% light, no water). Each point represents one seedling. Lines are best‐fit linear models for each treatment with a common intercept, and NSC concentrations are ln transformed. Models include seedlings from a nonsignificant defoliation treatment that is pooled with other treatments.

Although NSC concentrations decreased in many instances, there was still a substantial portion of the NSC present. When significant, across species the decrease from the starting value to 7 weeks ranged from 22 to 60% in the shade + drought treatment, 14 to 17% in the drought treatment, and 13 to 44% in the shade treatment. The greatest decrease was in the shade + drought treatment in *A. rubrum*, which after seven weeks lost 60% of its initial NSC concentration (Fig. 4).

## Discussion

All five species of temperate deciduous tree seedlings stopped accumulating both biomass and total NSC while under shade and drought. This supports our first hypothesis that severe shade and drought are carbon limiting. Furthermore, we observed depletion of stored carbon in all species under combined shade and drought, suggesting that seedlings were utilizing reserves in response to the carbon limitation imposed by the most extreme stress treatment, which is consistent with our second hypothesis.

Overall, our results indicate that stress decreases total NSC reserves and that seedlings rely on NSC to overcome the carbon limitation imposed by stress. However, species had some interesting differences in NSC response to shade and drought alone, and patterns depended both on the species and the type of stress, somewhat consistent with our third hypothesis, although NSC components had varying patterns across species. Our findings also highlight the importance of looking at both starch and soluble sugars separately for understanding the multiple roles of carbon reserves in stress tolerance.

All species depleted NSC under the harshest treatment with shade + drought, and there were no cases of total NSC increasing in either shade or drought alone. The NSC decline in the shade + drought treatment for all species suggests that the ability to maintain NSC reserves is lost under both stresses. In addition, NSC decreased at the greatest rate in the harshest treatment of shade + drought for all species, indicating that there is an additive effect. This could indicate that carbon becomes even more limited under both stresses or that seedlings lose their ability to maintain carbon stores.

The drought‐tolerant oaks were able to maintain their overall NSC reserves while under a single stress, but total NSC does not tell the entire story. Starch made up a majority of total NSC, but starch and soluble sugars responded to stress differently across species. In drought, total starch decreased in every species, but in the more drought‐tolerant species (*Q. rubra*,* Q. velutina, and A. rubrum*) the decrease was offset by an increase in soluble sugars, leading to no change in total NSC. These results are consistent with the idea that NSC is used for something other than metabolic demand, such as a source of solutes that help to regulate osmotic balance and maintain water potentials during drought (Nardini et al. 2011; Secchi and Zwieniecki 2011; O'Brien et al. 2014). The ability to mobilize NSC for these functions could influence tolerance to drought (Sala et al. 2010), which may be an explanation for why the decrease in the most drought intolerant *B. papyrifera* and intermediate *F. americana* were not able increase soluble sugars. The least drought‐tolerant species (*B. papyrifera* and *F. americana)* were also not able to maintain total NSC in drought in contrast to the other species. At the same time, all species depleted starch reserves during drought. The more drought‐tolerant species may convert starch to soluble sugars, possibly to alter osmotic balance, while maintaining total NSC. In contrast, drought intolerant species decreased total NSC, suggesting that drought intolerant species are more likely to die from carbon starvation.

Unlike in drought, soluble sugars did not increase in shade alone, but rather maintained concentrations in all species except for *B. papyrifera*. The least shade‐ and drought‐tolerant *B. papyrifera* and *F. americana* experienced decreased soluble sugars in shade + drought, and *B. papyrifera* in just shade. Drought‐tolerant species, such as the oaks, may have lower respiratory demands to or be better at down regulating these sinks, which could explain the lack of NSC response in either under shade or drought alone.

Species differences in initial NSC reserves generally followed species' drought tolerance rankings. The least shade tolerant *B. papyrifera* had the lowest NSC, the most shade tolerant *A. rubrum* had intermediate NSC. The amount of NSC was greater for the slower growing and more drought‐tolerant oaks, and lower in fast growing and drought and shade intolerant *B. papyrifera*. The oaks likely have lower respiratory demand and could potentially respond to shade by down regulating to maintain their NSC reserves. The shade treatment was not strong enough on its own to significantly deplete the large stores of carbohydrates in the oaks. While active maintenance of carbon reserves could counteract depletion through a shift from growth to storage as suggested in some other studies (Smith and Stitt 2007; Gibon et al. 2009), this is unlikely as photosynthesis likely would not be possible at extremely low light levels, although we did not measure gas exchange directly. In the species with the lowest initial NSC. *A. rubrum, B. papyrifera*, and *F. americana*, we saw a decrease in total NSC and starch in the shade treatment.

Defoliation had little effect on NSC for any species, although there was a nonsignificant tendency for defoliated seedlings to have a slower increase in both biomass and NSC. Regardless, 50% defoliation had weak effects, which suggests compensatory photosynthetic responses. Similar results have been found in other studies, where partial leaf removal had negligible effects on seedling NSC, while stronger effects were found under complete leaf removal (Canham et al. 1999). In contrast, NSC often decreases with partial defoliation in larger trees (Eyles et al. 2009; Landhäusser and Lieffers 2011; Quentin et al. 2011), and NSC pools in defoliated trees can be replenished to greater than original levels under high light (Van Der Heyden and Stock 1996). NSC depletion is likely dependent on both the level of defoliation and the ontogenetic stage of the tree as trees tend to store more carbohydrates as they get larger.

Our results suggest that under sever shade and drought, NSC reserves are utilized under carbon‐limiting stress and may play a role in survival. While our treatments were severe and seedlings were presumably close to experiencing mortality, none was observed. It also is important to point out that there were no instances of complete reserve depletion in any of the treatments, despite NSC concentrations reaching very low levels in some cases (0.25% for *B. papyrifera* in the shade + drought treatment). NSC dynamics observed here are consistent with other studies where carbohydrate reserves are mobilized, but not completely depleted (Millard et al. 2007; Sala et al. 2010). Our snapshot of seedling NSC may not be generalizable to adult trees where mortality after drought can take decades (Sala et al. 2010). Lastly, to obtain a full picture of carbon balance, respiration rates and other sinks need to be tested concurrently. This is especially important because increased drought is usually accompanied by an increase in temperature, which can increase respiration rates (Hartley et al. 2006; Adams et al. 2009). Thus, long‐term studies that link NSC dynamics with carbon sources and sinks as well as mortality events are needed. Using seedlings, we were able to construct a whole plant view of carbohydrates and show how NSC pools, concentrations and total biomass change. We also show that species have different responses to various types of stress and that the interaction of stress types may be important for understanding differences among species.

## Conflict of Interest

None declared.

## Data Accessibility

Data from the manuscript will be submitted at DRYAD.

## Supporting information


**Figure S1.** Relationship between total plant mass and nonstructural carbohydrate (NSC) concentrations, stem and root NSC concentrations.
**Figure S2.** Parameter estimates for linear models of plant mass as a function of time under five stress treatments for five temperate tree species.
**Figure S3.** Parameter estimates for linear models of nonstructural carbohydrate (NSC) concentrations (soluble sugars + starch) in the stem and root of seedlings as a function of time under five stress treatments for five temperate tree species.
**Figure S4.** Parameter estimates for linear models of starch as a function of time under five stress treatments for five temperate tree species.
**Figure S5.** Parameter estimates for linear models of soluble sugars as a function of time under five stress treatments for five temperate tree species.

## References

[ece31819-bib-0001] Adams, H. D. , M. Guardiola‐Claramonte , G. A. Barron‐Gafford , J. C. Villegas , D. D. Breshears , C. B. Zou , et al. 2009 Temperature sensitivity of drought‐induced tree mortality portends increased regional die‐off under global‐change‐type drought. Proc. Natl Acad. Sci. USA 106:7063–7066. 19365070 10.1073/pnas.0901438106 PMC2678423

[ece31819-bib-0002] Adams, H. D. , M. J. Germino , D. D. Breshears , G. A. Barron‐Gafford , M. Guardiola‐Claramonte , C. B. Zou , et al. 2013 Nonstructural leaf carbohydrate dynamics of *Pinus edulis* during drought‐induced tree mortality reveal role for carbon metabolism in mortality mechanism. New Phytol. 197:1142–1151. 23311898 10.1111/nph.12102

[ece31819-bib-0003] Allen, C. D. , A. K. Macalady , H. Chenchouni , D. Bachelet , N. G. McDowell , M. Vennetier , et al. 2010 A global overview of drought and heat‐induced tree mortality reveals emerging climate change risks for forests. For. Ecol. Manage. 259:660–684.

[ece31819-bib-0004] Anderegg, W. R. L. , J. A. Berry , D. D. Smith , J. S. Sperry , L. D. L. Anderegg , and C. B. Field . 2012 The roles of hydraulic and carbon stress in a widespread climate‐induced forest die‐off. Proc. Natl Acad. Sci. USA 109:233–237. 22167807 10.1073/pnas.1107891109 PMC3252909

[ece31819-bib-0005] Bolker, B. 2012 bbmle R package. R Development Core Team. Version 1.0.5.2. R Foundation for Statistical Computing, Vienna, Austria. Available at http://www.r-project.org/.

[ece31819-bib-0006] Bréda, N. , R. Huc , A. Granier , and E. Dreyer . 2006 Temperate forest trees and stands under severe drought: a review of ecophysiological responses, adaptation processes and long‐term consequences. Ann. For. Sci. 63:625–644.

[ece31819-bib-0007] Breshears, D. D. , O. B. Myers , C. W. Meyer , F. J. Barnes , C. B. Zou , C. D. Allen , et al. 2009 Tree die‐off in response to global change‐type drought, mortality insights from a decade of plant water potential measurements. Front. Ecol. Environ. 7:185–189.

[ece31819-bib-0008] Burnham, K. P. , and D. R. Anderson . 2002 Model Selection and Multimodel Inference: A Practical Information‐Theoretic Approach, 2nd edn Springer, New York.

[ece31819-bib-0009] Burns, R. M. , and Honkala B. H. 1990 Silvics of North America: Volume 2. Hardwoods. Agriculture Handbook 654. U.S. Department of Agriculture, Forest Service, Washington, DC.

[ece31819-bib-0010] Canham, C. D. , R. K. Kobe , E. F. Latty , and R. L. Chazdon . 1999 Interspecific and intraspecific variation in tree seedling survival: effects of allocation to roots versus carbohydrate reserves. Oecologia 121:1–11. 10.1007/s004420050900 28307877

[ece31819-bib-0011] Carnicer, J. , M. Coll , M. Ninyerola , X. Pons , G. Sánchez , and J. Peñuelas . 2011 Widespread crown condition decline, food web disruption, and amplified tree mortality with increased climate change‐type drought. Proc. Natl Acad. Sci. USA 108:1474–1478. 21220333 10.1073/pnas.1010070108 PMC3029725

[ece31819-bib-0012] Chapin, F. S. , E.‐D. Schulze , and H. A. Mooney . 1990 The ecology and economics of storage in plants. Annu. Rev. Ecol. Syst. 21:423–447.

[ece31819-bib-0013] Chapin, F. S. , K. Autumn , and F. Pugnaire . 1993 Evolution of suites of traits in response to environmental stress. Am. Nat. 142:S78–S92.

[ece31819-bib-0014] Choat, B. , S. Jansen , T. J. Brodribb , H. Cochard , S. Delzon , R. Bhaskar , et al. 2012 Global convergence in the vulnerability of forests to drought. Nature 491:752–756. 23172141 10.1038/nature11688

[ece31819-bib-0015] Dietze, M. C. , A. Sala , M. S. Carbone , C. I. Czimczik , J. A. Mantooth , A. D. Richardson , et al. 2014 Nonstructural carbon in woody plants. Annu. Rev. Plant Biol. 65:667–687. 24274032 10.1146/annurev-arplant-050213-040054

[ece31819-bib-0016] DuBois, M. , K. A. Gilles , J. K. Hamilton , P. A. Rebers , and F. E. Smith . 1956 Colorimetric method for determination of sugars and related substances. Anal. Chem. 28:350–356.

[ece31819-bib-0017] Eyles, A. , E. A. Pinkard , and C. Mohammed . 2009 Shifts in biomass and resource allocation patterns following defoliation in *Eucalyptus globulus* growing with varying water and nutrient supplies. Tree Physiol. 29:753–764. 19324694 10.1093/treephys/tpp014

[ece31819-bib-0018] Fisher, R. , N. G. McDowell , D. Purves , P. Moorcroft , S. Sitch , P. Cox , et al. 2010 Assessing uncertainties in a second‐generation dynamic vegetation model caused by ecological scale limitations. New Phytol. 187:666–681. 20618912 10.1111/j.1469-8137.2010.03340.x

[ece31819-bib-0019] Galiano, L. , J. Martínez‐Vilalta , and F. Lloret . 2011 Carbon reserves and canopy defoliation determine the recovery of Scots pine 4 yr after a drought episode. New Phytol. 190:750–759. 21261625 10.1111/j.1469-8137.2010.03628.x

[ece31819-bib-0020] Galvez, D. A. , S. M. Landhäusser , and M. T. Tyree . 2011 Root carbon reserve dynamics in aspen seedlings: does simulated drought induce reserve limitation? Tree Physiol. 31:250–257. 21444372 10.1093/treephys/tpr012

[ece31819-bib-0021] Gibon, Y. , E. T. Pyl , R. Sulpice , J. E. Lunn , M. Höhne , M. Günther , et al. 2009 Adjustment of growth, starch turnover, protein content and central metabolism to a decrease of the carbon supply when Arabidopsis is grown in very short photoperiods. Plant Cell Environ. 32:859–874. 19236606 10.1111/j.1365-3040.2009.01965.x

[ece31819-bib-0022] Gruber, A. , D. Pirkebner , C. Florian , and W. Oberhuber . 2011 No evidence for depletion of carbohydrate pools in Scots pine (*Pinus sylvestris* L.) under drought stress. Plant Biol. 14:142–148. 21974742 10.1111/j.1438-8677.2011.00467.x PMC3427021

[ece31819-bib-0023] Hartley, I. P. , A. F. Armstrong , R. Murthy , G. Barron‐Gafford , P. Ineson , and O. K. Atkin . 2006 The dependence of respiration on photosynthetic substrate supply and temperature: integrating leaf, soil and ecosystem measurements. Glob. Change Biol. 12:1954–1968.

[ece31819-bib-0025] Hoch, G. , A. Richter , and C. Körner . 2003 Non‐structural carbon compounds in temperate forest trees. Plant Cell Environ. 26:1067–1081.

[ece31819-bib-0026] Kitajima, K. 1994 Relative importance of photosynthetic traits and allocation patterns as correlates of seedling shade tolerance of 13 tropical trees. Oecologia 98:419–428. 10.1007/BF00324232 28313920

[ece31819-bib-0027] Kobe, R. K. 1997 Carbohydrate allocation to storage as a basis of interspecific variation in sapling survivorship and growth. Oikos 80:226–233.

[ece31819-bib-0028] Kobe, R. K. , M. Iyer , and M. B. Walters . 2010 Optimal partitioning theory revisited: nonstructural carbohydrates dominate root mass responses to nitrogen. Ecology 91:166–179. 20380206 10.1890/09-0027.1

[ece31819-bib-0030] Kozlowski, T. T. 1992 Carbohydrate sources and sinks in woody plants. Bot. Rev. 58:107–222.

[ece31819-bib-0031] Landhäusser, S. M. , and V. J. Lieffers . 2011 Defoliation increases risk of carbon starvation in root systems of mature aspen. Trees 26:653–661.

[ece31819-bib-0032] Machado, R. A. R. , A. P. Ferrieri , C. A. M. Robert , G. Glauser , M. Kallenbach , I. T. Baldwin , et al. 2013 Leaf‐herbivore attack reduces carbon reserves and regrowth from the roots via jasmonate and auxin signaling. New Phytol. 200:1–13. 23914830 10.1111/nph.12438

[ece31819-bib-0033] McDowell, N. G. 2011 Mechanisms linking drought, hydraulics, carbon metabolism, and vegetation mortality. Plant Physiol. 155:1051–1059. 21239620 10.1104/pp.110.170704 PMC3046567

[ece31819-bib-0034] McDowell, N. G. , W. T. Pockman , C. D. Allen , D. D. Breshears , N. Cobb , T. Kolb , et al. 2008 Mechanisms of plant survival and mortality during drought, why do some plants survive while others succumb to drought? New Phytol. 178:719–739. 18422905 10.1111/j.1469-8137.2008.02436.x

[ece31819-bib-0035] Meier, I. C. , and C. Leuschner . 2008 Belowground drought response of European beech: fine root biomass and carbon partitioning in 14 mature stands across a precipitation gradient. Glob. Change Biol. 14:2081–2095.

[ece31819-bib-0036] Millard, P. , M. Sommerkorn , and G. Grelet . 2007 Environmental change and carbon limitation in trees: a biochemical, ecophysiological and ecosystem appraisal. New Phytol. 175:11–28. 17547663 10.1111/j.1469-8137.2007.02079.x

[ece31819-bib-0037] Mitchell, P. J. , A. P. O'Grady , D. T. Tissue , D. A. White , M. L. Ottenschlaeger , and E. A. Pinkard . 2013 Drought response strategies define the relative contributions of hydraulic dysfunction and carbohydrate depletion during tree mortality. New Phytol. 197:862–872. 23228042 10.1111/nph.12064

[ece31819-bib-0038] Muller, B. , F. Pantin , M. Génard , O. Turc , S. Freixes , M. Piques , et al. 2011 Water deficits uncouple growth from photosynthesis, increase C content, and modify the relationships between C and growth in sink organs. J. Exp. Bot. 62:1715–1729. 21239376 10.1093/jxb/erq438

[ece31819-bib-0039] Myers, J. A. , and K. Kitajima . 2007 Carbohydrate storage enhances seedling shade and stress tolerance in a neotropical forest. J. Ecol. 95:383–395.

[ece31819-bib-0040] Nardini, A. , M. A. Lo Gullo , and S. Salleo . 2011 Refilling embolized xylem conduits: is it a matter of phloem unloading? Plant Sci. 180:604–611. 21421408 10.1016/j.plantsci.2010.12.011

[ece31819-bib-0041] O'Brien, M. J. , S. Leuzinger , C. D. Philipson , J. Tay , and A. Hector . 2014 Drought survival of tropical tree seedlings enhanced by non‐structural carbohydrate levels. Nat. Clim. Chang. 4:1–5.

[ece31819-bib-0042] Piper, F. I. , M. Reyes‐Díaz , L. J. Corcuera , and C. H. Lusk . 2009 Carbohydrate storage, survival, and growth of two evergreen *Nothofagus* species in two contrasting light environments. Ecol. Res. 24:1233–1241.

[ece31819-bib-0043] Poorter, L. , and K. Kitajima . 2007 Carbohydrate storage and light requirements of tropical moist and dry forest tree species. Ecology 88:1000–1011. 17536715 10.1890/06-0984

[ece31819-bib-0044] Quentin, A. G. , C. L. Beadle , A. P. O'Grady , and E. A. Pinkard . 2011 Effects of partial defoliation on closed canopy *Eucalyptus globulus* Labilladière: growth, biomass allocation and carbohydrates. For. Ecol. Manage. 261:695–702.

[ece31819-bib-0045] R Development Core Team . 2010 R: A Language and Environment for Statistical Computing. R Foundation for Statistical Computing, Vienna, Austria Available at http://www.R-project.org/.

[ece31819-bib-0046] Sack, L. , and P. J. Grubb . 2002 The combined impacts of deep shade and drought on the growth and biomass allocation of shade‐tolerant woody seedlings. Oecologia 131:175–185. 10.1007/s00442-002-0873-0 28547684

[ece31819-bib-0047] Sala, A. , F. Piper , and G. Hoch . 2010 Physiological mechanisms of drought‐induced tree mortality are far from being resolved. New Phytol. 186:274–281. 20409184 10.1111/j.1469-8137.2009.03167.x

[ece31819-bib-0048] Sala, A. , D. R. Woodruff , and F. C. Meinzer . 2012 Carbon dynamics in trees: feast or famine? Tree Physiol. 32:764–775. 22302370 10.1093/treephys/tpr143

[ece31819-bib-0049] Secchi, F. , and M. A. Zwieniecki . 2011 Sensing embolism in xylem vessels: the role of sucrose as a trigger for refilling. Plant Cell Environ. 34:514–524. 21118423 10.1111/j.1365-3040.2010.02259.x

[ece31819-bib-0050] Sevanto, S. , N. G. McDowell , L. T. Dickman , R. Pangle , and W. T. Pockman . 2014 How do trees die? A test of the hydraulic failure and carbon starvation hypotheses. Plant Cell Environ. 37:153–161. 23730972 10.1111/pce.12141 PMC4280888

[ece31819-bib-0051] Smith, A. M. , and M. Stitt . 2007 Coordination of carbon supply and plant growth. Plant Cell Environ. 30:1126–1149. 17661751 10.1111/j.1365-3040.2007.01708.x

[ece31819-bib-0052] Van Der Heyden, F. , and W. D. Stock . 1996 Regrowth of a semiarid shrub following simulated carbon browsing: the role of reserve carbon. Funct. Ecol. 10:647–653.

[ece31819-bib-0053] Williams, A. P. , C. D. Allen , C. I. Millar , T. W. Swetnam , J. Michaelsen , C. J. Still , et al. 2010 Forest responses to increasing aridity and warmth in the southwestern United States. Proc. Natl Acad. Sci. USA 107:21289–21294. 21149715 10.1073/pnas.0914211107 PMC3003095

