# Supplementary material for: Drought and shade deplete nonstructural carbohydrate reserves in seedlings of five temperate tree species
Source: Ecol Evol. 2015 Nov 19;5(23):5711–21. doi: 10.1002/ece3.1819 (PMC4813112; doi:10.1002/ece3.1819)
Supplement: Supplementary file 1 — Figure S1. Relationship between total plant mass and nonstructural carbohydrate (NSC) concentrations, stem and root NSC concentrations. Figure S2. Parameter estimates for linear models of plant mass as a function of time under five stress treatments for five temperate tree species. Figure S3. Parameter estimates for linear models of nonstructural carbohydrate (NSC) concentrations (soluble sugars + starch) in the stem and root of seedlings as a function of time under five stress treatments for five temperate tree species. Figure S4. Parameter estimates for linear models of starch as a function of time under five stress treatments for five temperate tree species. Figure S5. Parameter estimates for linear models of soluble sugars as a function of time under five stress treatments for five temperate tree species. [file ECE3-5-5711-s001.docx]

**Supporting Information**

**Figure S1**

Relationship between total plant mass and nonstructural carbohydrate (NSC) concentrations, stem and root NSC concentrations.

**Figure S2**

Parameter estimates for linear models of plant mass as a function of time under five treatments: C = control (50 % light, well-watered), D = drought (50 % light and no water), S = shade (< 3 % light, well-watered), SD = shade + drought (< 3 % light, no water) for five temperate tree species. Parameters include a common intercept with different slopes for each treatment and 95 % support intervals (SI), and plant mass is natural log transformed. Models include seedlings from a non-significant defoliation treatment that is pooled with other treatments.

**Figure S3**

Parameter estimates for linear models of nonstructural carbohydrate (NSC) concentrations (soluble sugars + starch) in the stem and root of seedlings as a function of time under five treatments: C = control (50 % light, well-watered), D = drought (50 % light and no water), S = shade (< 3 % light, well-watered), SD = shade + drought (< 3 % light, no water) for five temperate tree species. Parameters include a common intercept with different slopes for each treatment and 95 % support intervals (SI), and NSC concentrations are natural log transformed. Models include seedlings from a non-significant defoliation treatment that is pooled with other treatments.

**Figure S4**

Parameter estimates for linear models of starch as a function of time under five treatments: C = control (50 % light, well-watered), D = drought (50 % light and no water), S = shade (< 3 % light, well-watered), SD = shade + drought (< 3 % light, no water) for five temperate tree species. Parameters include a common intercept with different slopes for each treatment and 95 % support intervals (SI) and starch concentrations are natural log transformed. Models include seedlings from a non-significant defoliation treatment that is pooled with other treatments.

**Figure S5**

Parameter estimates for linear models of soluble sugars as a function of time under five treatments: C = control (50 % light, well-watered), D = drought (50 % light and no water), S = shade (< 3 % light, well-watered), SD = shade + drought (< 3 % light, no water) for five temperate tree species. Parameters include a common intercept with different slopes for each treatment and 95 % support intervals (SI) and soluble sugar concentrations are natural log transformed. Models include seedlings from a non-significant defoliation treatment that is pooled with other treatments.
